# Clinical and laboratory characteristics of patients hospitalized with severe COVID-19 in New Orleans, August 2020 to September 2021

**DOI:** 10.1038/s41598-024-57306-5

**Published:** 2024-03-19

**Authors:** Arnaud Drouin, Ian D. Plumb, Matthew McCullough, Jade James Gist, Sharon Liu, Marc Theberge, Joshua Katz, Matthew Moreida, Shelby Flaherty, Bhoomija Chatwani, Melissa Briggs Hagen, Claire M. Midgley, Dahlene Fusco

**Affiliations:** 1 Department of Medicine, Tulane University School of Medicine, 1430 Tulane Avenue, New Orleans, LA 70130 USA; 2 University Medical Center, New Orleans, LA USA; 3 Applied Epidemiology Studies Team, Epidemiology Branch, and on detail to the Global Respiratory Viruses Branch Coronavirus and Other Respiratory Viruses Division, Centers for Disease Control, Atlanta, GA USA; 4 Eagle Health Analytics, Atlanta, GA USA; 5 School of Public Health and Tropical Medicine, Tulane University, New Orleans, LA USA

**Keywords:** COVID-19, SARS CoV-2, qRT-PCR, Anti-N antibody, Viral infection, Epidemiology

## Abstract

Louisiana experienced high morbidity and mortality from COVID-19. To assess possible explanatory factors, we conducted a cohort study (ClinSeqSer) of patients hospitalized with COVID-19 in New Orleans during August 2020–September 2021. Following enrollment, we reviewed medical charts, and performed SARS-CoV-2 RT-PCR testing on nasal and saliva specimens. We used multivariable logistic regression to assess associations between patient characteristics and severe illness, defined as ≥ 6 L/min oxygen or intubation. Among 456 patients, median age was 56 years, 277 (60.5%) were Black non-Hispanic, 436 (95.2%) had underlying health conditions, and 358 were unvaccinated (92.0% of 389 verified). Overall, 187 patients (40.1%) had severe illness; 60 (13.1%) died during admission. In multivariable models, severe illness was associated with age ≥ 65 years (OR 2.08, 95% CI 1.22–3.56), hospitalization > 5 days after illness onset (OR 1.49, 95% CI 1.01–2.21), and SARS CoV-2 cycle threshold (Ct) result of < 32 in saliva (OR 4.79, 95% CI 1.22–18.77). Among patients who were predominantly Black non-Hispanic, unvaccinated and with underlying health conditions, approximately 1 in 3 patients had severe COVID-19. Older age and delayed time to admission might have contributed to high case-severity. An association between case-severity and low Ct value in saliva warrants further investigation.

## Introduction

COVID-19 continues to have a broad clinical spectrum that encompasses a range of acute and longer-term complications^1,2^. Although overall severity of COVID-19 has become milder since the beginning of the COVID-19 pandemic^3^, some patients continue to experience severe illness. Louisiana has experienced high morbidity and mortality during the COVID-19 pandemic compared with other regions^4^, especially among Black non-Hispanic persons, and among those with underlying health conditions^5^.

Several potential reasons may explain the relatively severe disease in Louisiana. First, impacts of the pandemic have highlighted underlying inequalities^6^ that may lead to the accumulation of specific comorbidities. Advanced age, male gender and certain comorbidities such as obesity, diabetes mellitus, cardiovascular disease, chronic lung disease and kidney disease have been identified as risk factors for severe outcomes^7–9^. Louisiana has the second highest prevalence of adults diagnosed with diabetes in the United States, at 12.9%, and has the highest rate of newly diagnosed adults with diabetes among states in the continental U.S^10^. Prevalence of self-reported obesity in Louisiana is 38.6%, ranked 43 of 50 for worst rates of obesity among U.S. states^11^. Second, inequalities might have been perpetuated by delayed access to care^5^. Third, severe illness might reflect virologic factors, although this might explain temporal rather than geographic differences in severity^12–14^. Initial variants of SARS-CoV-2 led to more severe illness than since predominance by the Omicron variant^13^, and lower PCR cycle threshold (Ct) values (which correlate with higher SARS-CoV-2 viral load) are associated with adverse outcomes among patients hospitalized with COVID-19^15–19^. Fourth, host immunity plays an important role in attenuating severity, whether following infection or vaccination. Populations that were both SARS-CoV-2 naïve and unvaccinated were therefore at particular risk of severe outcomes. Finally, not receiving recommended treatment might have led to more severe illness.

To assess the potential contributions of these factors to severe outcomes among hospitalized patients in Louisiana, we conducted a cohort study among patients hospitalized with COVID-19 during August 2020–September 2021. Following enrollment, we reviewed medical charts, performed SARS-CoV-2 RT-PCR testing on nasal and saliva specimens, and measured SARS-CoV-2 anti-nucleocapsid antibody titers in available serum specimens. We described a range of demographic, clinical and laboratory characteristics among patients hospitalized with COVID-19 during the study period. Using multivariable logistic regression, we then assessed the association between patient characteristics and severe illness, which we defined for analysis purposes as receiving ≥ 6 L/min of supplemental oxygen, intubation or inpatient death.

## Results

### Participants included

**Figure 1:**
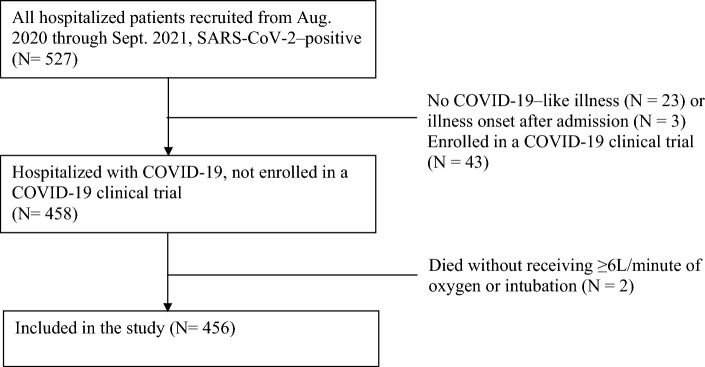
Participants included in the study.

Among 527 patients who were enrolled from August 2020 until September 2021, 456 were included in the analysis; among 458 patients hospitalized with COVID-19-like illness, two patients had died without receiving ≥ 6 L/minute of supplementary oxygen—one patient with atrial fibrillation and hypertension, and another with several comorbidities including chronic myelogenous leukemia with splenectomy and hepatic cirrhosis. We excluded these patients a priori because of other possible causes of death (Fig. 1). Overall, characteristics of the remaining 456 patients who were included in the analysis were similar in age, gender, and ethnicity to those of all inpatients diagnosed with COVID-19 at participating health facilities during the same period, although those enrolled more frequently had Black race (60.5% vs 50.3%) and non-Hispanic ethnicity (96.7% vs. 81.3%; Supplementary Table 1). Among patients enrolled in the study, characteristics of the 456 patients included were generally similar to those of patients who were excluded (Supplementary Table 2).

### Demographic and clinical characteristics

Among the 456 hospitalized patients, illness onset was most frequent before Delta predominance (n = 258, 56.6%), the median age was 56 years (range 18–98), 259 (56.8%) were male and 275 (60.3%) were Black non-Hispanic (Table 1). Of 434 patients (95.2%) with at least one underlying health condition, the most prevalent were cardiovascular conditions including hypertension (n = 279, 61.2%), obesity (n = 40.4%, 184) and diabetes mellitus (n = 33.8%, 154); 370 patients (81.1%) had multiple underlying health conditions. Patients were admitted with COVID-19 a median of 5 days (range 2 to 9) after symptom onset. At symptom onset, 388 patients had known vaccination status, of whom 31 (8.0%) had received at least one COVID-19 vaccine dose. Treatments administered included dexamethasone (n = 293, 64.3%), remdesivir (n = 198, 43.4%), anti-spike monoclonal antibody therapy (n = 11, 2.4%), baricitinib (n = 2, 0.4%), tocilizumab (n = 17, 3.7%), and convalescent plasma (n = 3, 0.7%).

**Table 1:** Background characteristics of study participants by severity of COVID-19.

Total	Overall	Non-Severe	Severe^a^	*P* value^b^
	N = 456	N = 269	N = 187	
Age group, years Range: [18–98] Median: 56				0.036
18–44	112 (24.6%)	76 (28.3%)	36 (19.3%)	
45–64	214 (46.9%)	126 (46.8%)	88 (47.1%)	
≥ 65	130 (28.5%)	67 (24.9%)	63 (33.7%)	
Sex				0.592
Female	197 (43.2%)	119 (44.2%)	78 (41.7%)	
Male	259 (56.8%)	150 (55.8%)	109 (58.3%)	
Race and ethnicity				0.145
Black, non-Hispanic	275 (60.3%)	169 (62.8%)	106 (56.7%)	
White, non-Hispanic	151 (33.1%)	88 (32.7%)	63 (33.7%)	
Hispanic	14 (3.1%)	5 (1.9%)	9 (4.8%)	
Other, non-Hispanic^c^	16 (3.5%)	7 (2.6%)	9 (4.8%)	
Any underlying health conditions^d^				0.188
0	22 (4.8%)	15 (5.6%)	7 (3.7%)	
1	64 (14.0%)	32 (11.9%)	32 (17.1%)	
2	75 (16.4%)	50 (18.6%)	25 (13.4%)	
> 2	295 (64.7%)	172 (63.9%)	123 (65.8%)	
Underlying health conditions				
Cardiac disease	279 (61.2%)	160 (59.5%)	119 (63.6%)	0.370
Pulmonary disease	108 (23.7%)	67 (24.9%)	41 (21.9%)	0.461
Neurologic disease	109 (23.9%)	69 (25.7%)	40 (21.4%)	0.294
Renal disease	76 (16.7%)	35 (13.0%)	41 (21.9%)	0.012
Liver disease	17 (3.7%)	8 (3.0%)	9 (4.8%)	0.308
Immunocompromised	93 (20.4%)	60 (22.3%)	33 (17.6%)	0.225
Diabetes mellitus	154 (33.8%)	89 (33.1%)	65 (34.8%)	0.710
Hematological disease	58 (12.7%)	38 (14.1%)	20 (10.7%)	0.279
Autoimmune disease	64 (14.0%)	35 (13.0%)	29 (15.5%)	0.450
Smoking history^e^	140 (30.7%)	97 (36.1%)	43 (23.0%)	0.003
Substance abuse history	88 (19.3%)	61 (22.7%)	27 (14.4%)	0.028
Obesity	184 (40.4%)	91 (33.8%)	93 (49.7%)	< 0.001
Endocrine disease	31 (6.8%)	18 (6.7%)	13 (7.0%)	0.913
Gastrointestinal disease	87 (19.1%)	55 (20.4%)	32 (17.1%)	0.373

^a^Received high-flow (“ ≥ 6 L/min) nasal cannula/face mask (not able to distinguish) or were intubated, including subjects who died following high-flow/intubation.

bPearson’s Chi-squared test.

^**c**^“Non-Hispanic Other” refers to participants self-identifying as Asian, American Indian, other, or unknown.

dRange [0–14]; All groups counted among “Underlying health conditions”.

eCurrent or previous tobacco use.

### Differences in clinical and demographic characteristics by severity

Overall, 187 patients (41.0%) received ≥ 6 L/min oxygen during hospitalization and were classified as having ‘severe illness’; this included 118 (63.1%) patients who were not intubated, and 69 (36.9%) patients who were intubated (Table 2). Among the 187 patients with severe illness, 60 (32.1%) subsequently died during their hospital stay, 39 (20.9%) of whom died during the first 28 days after symptom onset. Patients with severe illness were admitted to hospital for a median 12 days (range 0–88) whereas those classified as non-severe were admitted for a median of 4 days (range 0–48).

**Table 2:** Clinical characteristics, by severity.

	Overall (N = 456)	Non-severe (N = 269)	Severe (N = 187)	*P* value
Days from illness onset to admission^a^				**0.020**
0–2	136/456 (29.8%)	93/269 (34.6%)	43/187 (23.0%)	
3–5	108/456 (23.7%)	63/269 (23.4%)	45/187 (24.1%)	
> 5	212/456 (46.5%)	113/269 (42.0%)	99/187 (52.9%)	
Variant predominance during illness onset^b^				0.134
Pre-delta	258/456 (56.6%)	160/269 (59.5%)	98/187 (52.4%)	
Delta	198/456 (43.4%)	109/269 (40.5%)	89/187 (47.6%)	
Vaccination status^c^				0.223
Unvaccinated	357/456 (78.3%)	216/269 (80.3%)	141/187 (75.4%)	
Received one mRNA dose	6/456 (1.3%)	2/269 (0.7%)	4/187 (2.1%)	
Completed ≥ 2 mRNA doses or ≥ 1 J&J dose ≥ 14 days prior	24/456 (5.3%)	16/269 (5.9%)	8/187 (4.3%)	
Received 3rd mRNA dose ≥ 14 days prior	1/456 (0.2%)	1/269 (0.4%)	0/187 (0.0%)	
Unknown	68/456 (14.9%)	34/269 (12.6%)	34/187 (18.2%)	
Dexamethasone				**< 0.001**
Not received	163/456 (35.7%)	144/269 (53.5%)	19/187 (10.2%)	
0–7 days after onset	176/456 (38.6%)	76/269 (28.3%)	100/187 (53.5%)	
8–14 days after onset	90/456 (19.7%)	35/269 (13.0%)	55/187 (29.4%)	
> 14 days after onset	27/456 (5.9%)	14/269 (5.2%)	13/187 (7.0%)	
Remdesivir				**< 0.001**
Not received	258/456 (56.6%)	184/269 (68.4%)	74/187 (39.6%)	
0–7 days after onset	114/456 (25.0%)	50/269 (18.6%)	64/187 (34.2%)	
8–14 days after onset	65/456 (14.3%)	25/269 (9.3%)	40/187 (21.4%)	
> 14 days after onset	19/456 (4.2%)	10/269 (3.7%)	9/187 (4.8%)	
Anti-spike monoclonal antibody^d^				0.631
Not received	445/456 (97.6%)	263/269 (97.8%)	182/187 (97.3%)	
0–7 days after onset	6/456 (1.3%)	3/269 (1.1%)	3/187 (1.6%)	
8–14 days after onset	4/456 (0.9%)	3/269 (1.1%)	1/187 (0.5%)	
> 14 days after onset	1/456 (0.2%)	0/269 (0.0%)	1/187 (0.5%)	
Anti-inflammatory monoclonal antibody (baricitinib, tocilizumab)				**< 0.001**
Not received	437/456 (95.8%)	269/269 (100%)	168/187 (89.8%)	
0–7 days after onset	6/456 (1.3%)	0/269 (0.0%)	6/187 (3.2%)	
8–14 days after onset	12/456 (2.6%)	0/269 (0.0%)	12/187 (6.4%)	
> 14 days after onset	1/456 (0.2%)	0/269 (0.0%)	1/187 (0.5%)	
Convalescent plasma				0.509
Not received	453/456 (99.3%)	268/269 (99.6%)	185/187 (98.9%)	
0–7 days after onset	2/456 (0.4%)	1/269 (0.4%)	1/187 (0.5%)	
8–14 days after onset	0/456 (%)	0/269 (0.0%)	0/187 (0.0%)	
> 14 days after onset	1/456 (0.2%)	0/269 (0.0%)	1/187 (0.5%)	
Respiratory support (mutually exclusive)^e,f,g^				**< 0.001**
Inpatient, no oxygen	134/456 (29.4%)	134/269 (49.8%)	N/A	
Oxygen, < 6L/min	135/456 (29.6%)	135/269 (50.2%)	N/A	
Oxygen, ≥ 6L/min (not intubated)	118/456 (25.9%)	N/A	118/187 (63.1%)	
Intubated	69/456 (15.1%)	N/A	69/187 (36.9%)	
Mortality^h^				
No death during hospitalization	396/456 (86.8%)	269/269 (100%)	127/187 (67.9%)	**N/A**
Death during hospitalization	60/456 (13.2%)	N/A	60/187 (32.1%)	**N/A**
Death within 28 days of symptom onset	39/456 (8.6%)	N/A	39/187 (20.9%)	**N/A**

a‘Illness’ as COVID-19–like symptoms: fever, chills, cough, shortness of breath, new loss of taste/smell, sore throat, fatigue, muscle or body aches, headache, diarrhea, headache, congestion, pressure in chest, new confusion, pale/gray/blue-colored skin and lips.

bDelta variant predominance defined as July 01, 2021, onwards.

cBy subgroup, the proportions completing ≥ 2 doses ≥ 14 days before illness onset were 4.0% if age < 65 years, 2.3% if ≥ 65 years, 1.3% if < 2 underlying health conditions, and 5% if ≥ 2 underlying health conditions.

dMonoclonal antibody therapies targeted against SARS-CoV-2 spike protein included casirivimab/imdevimab and bamlanivimab.

eDenotes highest level of respiratory support required during admission. Overall, 187 (41%) received high flow (≥ 6 L/min) or intubation, and were considered as severe for this analysis.

^f^When assessing maximum severity by respiratory support and mortality, 134 (29.4%) patients received no supplemental oxygen (all survived), 135 (29.6%) received low-flow oxygen (all survived), 107 (23.5%) received high-flow oxygen and survived, 20 (4.4%) were intubated and survived, and 60 (13.2%) died after receiving high-flow oxygen (n = 11) or intubation (n = 49).

^g^Median duration of admission was 4 days [range 0–48 days] for patients who were admitted but received no oxygen, 5 days [range 1–38 days] for those who received < 6 L/min of supplemental oxygen, 10 days [range 1–49 days] for those who received ≥ 6 L/min without intubation, and 21 days [range 0–88 days] for those who were intubated.

^h^Median duration of admission (until end of admission or inpatient death) was 17 days [range 0–88 days) among patients who survived, 17 days [range 0–59 days] among those who died in the hospital, and 12 days [range 0–28 days] among those who died during the 28 days after symptom onset.

Bold text indicates p values that reached statistical significance.

Compared with the 269 hospitalized patients who were classified as having ‘non-severe illness’, those with severe illness were more likely to be older, or to have obesity or renal disease, but were less likely to have a history of smoking or substance abuse (Table 1). Patients with severe illness were generally admitted later after symptom onset. Patients who were categorized as having severe illness were also more likely to have received dexamethasone, remdesivir, or other therapy at some point during admission (Table 2). Other characteristics were generally similar by severity (Tables 1 and 2).

In a multivariable model, patients with severe illness were more likely to be older than ≥ 65 years (OR 2.08, 95% confidence interval [CI] 1.22–3.56), admitted > 5 days after illness onset (OR 1.49, 1.01–2.21), and to have received COVID-19 therapy before receiving ≥ 6L/minute oxygen or intubation (OR 3.10, 1.99–4.83) (Table 3).

**Table 3:** Model of demographic and clinical characteristics associated with severity among patients admitted with COVID-19, August 2020–September 2021.

	No. severe/Total in category (% severe)	Unadjusted OR (95% CI)	Adjusted OR (95% CI)
Age group, years^a^	n/N (row %)		
18–44	36/112 (32.1%)	Ref	Ref
45–64	88/214 (41.1%)	1.47 (0.91–2.39)	1.52 (0.93–2.48)
≥ 65	63/130 (48.5%)	2.01 (1.19–3.40)	2.08 (1.22–3.56)
Sex^16^			
Female	78/198 (39.4%)	Ref	Ref
Male	109/260 (41.9%)	1.11 (0.76–1.62)	1.13 (0.77–1.66)
Race and ethnicity			
White, non-Hispanic	63/151 (41.7%)	Ref	Ref
Black, non-Hispanic	106/275 (38.6%)	0.87 (0.59–1.31)	0.94 (0.62–1.41)
Hispanic	9/14 (64.3%)	2.51 (0.80–7.86)	3.09 (0.97–9.81)
Other, non-Hispanic	9/16 (56.3%)	1.80 (0.64–5.08)	1.82 (0.64–5.18)
Underlying conditions			
0–1	39/86 (45.4%)	Ref	Ref
≥ 2	148/370 (40.0)	0.80 (0.50–1.29)	0.77 (0.46–1.27)
Vaccination status^b^			
Did not complete primary series^c^	179/431 (41.5%)	Ref	Ref
Completed primary series	8/25 (32.0%)	0.66 (0.28–1.57)	0.58 (0.24–1.41)
Variant predominance during illness onset			
Pre-delta (pre-July 1, 2021)	101/264 (38.3%)	Ref	Ref
Delta	86/192 (44.8%)	1.31 (0.90–1.91)	1.39 (0.94–2.06)
Time from symptom onset to hospital admission^d^			
0–5 days	88/244 (36.1%)	Ref	Ref
> 5 days	99/212 (46.7%)	1.53 (1.07–2.26)	1.49 (1.01–2.21)
Treatment within 0–7 days (if administered before outcome)^e^			
Any treatment^f^			
No	95/283 (33.6%)	Ref	Ref
Yes	92/173 (53.2%)	2.25 (1.53–3.29)	3.10 (1.99–4.83)
Dexamethasone			
No	99/292 (33.9%)	Ref	Ref
Yes	88/164 (53.7%)	2.26 (1.53–3.34)	3.07 (1.97–4.80)
Remdesivir			
No	135/354 (37.9%)	Ref	Ref
Yes	52/102 (51.0%)	1.69 (1.08–2.63)	2.05 (1.27–2.30)
Remdesivir or anti-spike monoclonal antibody^g^			
No	134/350 (38.1%)	Ref	Ref
Yes	53/106 (50.0%)	1.61 (1.04–2.50)	1.94 (1.22–3.11)
Dexamethasone or anti-inflammatory monoclonal antibody^hi^			3
No	99/292 (52.9%)	Ref	Ref
Yes	88/164 (47.1%)	2.26 (1.53–3.33)	3.07 (1.97–4.80)

^a^Adjusted for Age Group, Sex, Race/Ethnicity.

^b^Adjusted for Basic Demographics (includes Age Group, Sex, Race/Ethnicity, Comorbidities (≥ 2 vs. 0–1)).

^c^Considered as completed primary series if received 2 + mRNA or 1 + J&J ≥ 14 days before symptom onset; considered as not completed primary series if received < 2 mRNA doses < 14 days before symptom onset.

^d^Adjusted for Basic demographics and Vaccination.

^e^Adjusted for Basic demographics, Vaccination, Variant Period.

^f^Adjusted for Basic demographics, Vaccination, Variant Period, Symptom Onset to Hospital Admission.

^g^Receipt of dexamethasone, remdesivir, casirivimab/imdevimab, bamlanivimab, baricitinib, or tocilizumab.

^h^Monoclonal antibody therapies targeted against SARS-CoV-2 spike protein included casirivimab/imdevimab and bamlanivimab.

^i^We considered baricitinib and tocilizumab to be monoclonal antibodies with anti-inflammatory mechanisms.

### Cycle threshold (Ct) values

Overall, 361 patients (79.2%) had a Ct value reported from a nasal specimen (including 214 patients with a specimen collected before or without any administration of antiviral medication), and 318 patients (69.7%) had a Ct value reported from a saliva specimen (including 194 with a specimen collected before or without antiviral treatment); 291 (63.8%) had both nasal and saliva specimens. Compared with other patients enrolled, those with available Ct values were more likely to have mild disease, be Black non-Hispanic, and to be admitted before predominance of the Delta SARS-CoV-2 variant (Supplemental Table 3). Among patients with Ct values for specimens collected during the 0–7 days after illness onset, 69/155 (44.5%) of those with a nasal specimen and 63/142 (44.4%) of those with a saliva specimen had an initial Ct value < 32 (Table 4); Ct values tended to be higher when collected later from symptom onset, consistent with lower viral load later in clinical course (Supplementary Fig. 1).

**Table 4:** Laboratory results from specimens collected during hospitalization, by severity of COVID-19.

	Overall	Non-Severe	Severe	*P* value^a^
Patients with nasal swab result, collected 0–7 days after illness onset				
All patients				
Ct ≥ 32	86/155 (55.5%)	59/106 (55.7%)	27/49 (55.1%)	Ref
Ct < 32	69/155 (44.5%)	47/106 (44.3%)	22/49 (44.9%)	0.948
Patients with a result before or without ‘antiviral treatment’^b^				
Ct ≥ 32	50/101 (46.5%)	41/78 (52.6%)	9/23 (39.1%)	Ref
Ct < 32	51/101 (50.5%)	37/78 (47.4%)	14/23 (60.9%)	0.26
Patients with saliva result 0–7 days after onset				
All patients				
Ct ≥ 32	79/142 (55.6%)	66/102 (64.7%)	13/40 (32.5%)	Ref
Ct < 32	63/142 (44.4%)	36/102 (35.3%)	27/40 (67.5%)	< 0.001
Patients with a result before or without ‘antiviral treatment’				
Ct ≥ 32	60/95 (63.2%)	54/78 (69.2%)	6/17 (35.3%)	Ref
Ct < 32	35/95 (36.8%)	24/78 (30.8%)	11/17 (64.7%)	0.012
Patients with anti-nucleocapsid antibody test result, 0–14 days after onset^c^				
All patients				
Negative	48/125 (38.4%)	31/73 (42.5%)	17/52 (32.7%)	Ref
Positive	77/125 (61.6%)	42/73 (57.5%)	35/52 (67.3%)	0.269
Patients with anti-nucleocapsid antibody test result, 0–14 days after onset				
≤ 1.0 AU OD/mL^d^	65/125 (52.0%)	38/73 (52.0%)	27/52 (51.9%)	Ref
> 1.0 AU OD/mL	60/125 (48.0%)	35/73 (48.0%)	25/52 (48.1%)	0.988
Patients with anti-nucleocapsid antibody test result, 15–28 days after onset^e^				
All patients				
Negative	1/25 (4.0%)	1/14 (7.1%)	0/11 (0.0%)	Ref
Positive	24/25 (96.0%)	13/14 (92.9%)	11/11 (100.0%)	0.979
Patients with anti-nucleocapsid antibody test result, 15–28 days after onset				
≤ 43.5 AU OD/mL^f^	12/25 (48.0%)	9/14 (64.3%)	3/11 (27.3%)	Ref
> 43.5 AU OD/mL	13/25 (52.0%)	5/14 (35.7%)	8/11 (72.7%)	0.074

^a^Pearson’s Chi-squared test.

^b^Remdesivir, casirivimab/imdevimab, bamlanivimab/etesevimab; this includes specimens collected before any of these medications were received and includes specimens from those who did not receive any of these medications.

^c^Of 125 patients with antibody specimens collected 0–14 days post-symptom onset during hospitalization, 17 had repeat specimens. For patients with multiple specimens, the specimen with the highest OD value was recorded to indicate if ever positive or if OD was greater than the median for the time period.

^d^Median antibody level during 0–14 days post-symptom onset, and during hospitalization.

^e^Of 25 patients with antibody specimens collected 15–28 days post-symptom onset during hospitalization, 1 had repeat specimens. For the one patient with multiple specimens, the specimen with the highest OD value was recorded to indicate if ever positive or if OD was greater than median for the time period.

^f^Median antibody level during 15–28 days post-symptom onset, and during hospitalization.

**Figure 2:**
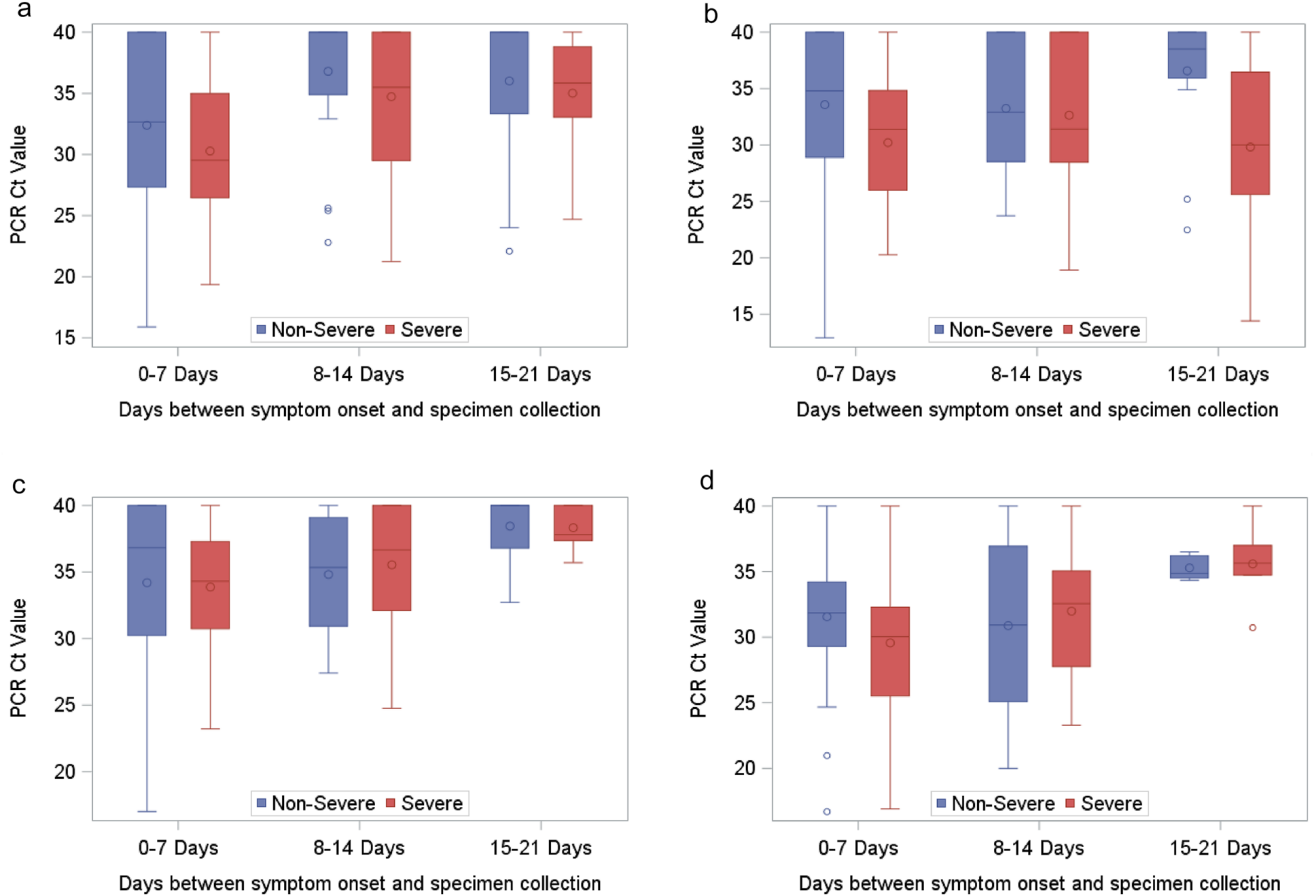
Ct values by specimen type, severity and time since onset. Distribution of PCR cycle threshold (Ct) values by time from symptom onset to PCR specimen collection. Panels **a** and **b** represent Ct values of nasal and saliva (respective) specimens collected before or without receipt of antiviral treatment (remdesivir, casirivimab/imdevimab, bamlanivimab/etesevimab). Panels **c** and **d** represent Ct values of nasal and saliva (respective) specimens collected after receipt of antiviral treatment (includes receipt of remdesivir, casirivimab/imdevimab, bamlanivimab/etesevimab).

Among patients with a Ct value result before or without any antiviral treatment, severe patients had lower Ct values than non-severe patients in saliva specimens collected during 0–7 days after illness onset (severe, 0–7 days: median (IQR) Ct = 31.5 (26.3–36.7); non-severe, 0–7 days: median (IQR) Ct = 35.1 (28.9–40.0); *p* = 0.015; Fig. 2). Correspondingly, severe patients were more likely than non-severe patients to have a Ct less than the median value of 32 in a saliva specimen collected 0–7 days post-onset and before or without receipt of antiviral treatment (Table 4). When specimens were collected after antiviral treatment, we observed no association with severity in either specimen type.

In a multivariable model, a Ct value < 32 in a saliva specimen collected during 0–7 days after onset was associated with severe illness if the specimen was collected before or in the absence of treatment (OR 4.79, 95% CI 1.22–18.77) but not if the specimen was collected after treatment (OR 1.96, 95% CI 0.51–7.53). By contrast nasal Ct value was not associated with severe illness (Table 5). These findings were similar for patients with nasal and saliva specimens collected on the same day, for specimens collected before the receipt of any oxygen, and for specimens collected during the 0–14 days after illness onset (Supplementary Table 4).

**Table 5:** Model of viral RNA cycle threshold (Ct) values during 0–7 days post symptom onset, associated with severity, August 2020–September 2021.

	No. severe/Total in category (% severe)	Unadjusted OR (95% CI)	Adjusted^a^ OR (95% CI)
Nasal specimen collected before or without antiviral treatment^b^			
Ct ≥ 32	9/50 (18.0%)	Ref	Ref
Ct < 32	14/51 (27.5%)	1.72 (0.67–4.45)	1.13 (0.38–3.41)
Nasal specimen collected after antiviral treatment			
Ct ≥ 32	18/36 (50.0%)	Ref	Ref
Ct < 32	8/18 (44.4%)	0.80 (0.26–2.49)	0.84 (0.22–3.21)
Saliva specimen collected before or without or without antiviral treatment			
Ct ≥ 32	6/60 (10.0%)	Ref	Ref
Ct < 32	11/35 (31.4%)	4.13 (1.37–12.45)	4.79 (1.22–18.77)
Saliva specimen collected after antiviral treatment			
Ct ≥ 32	7/19 (6.8%)	Ref	Ref
Ct < 32	16/28 (57.1%)	2.29 (0.69–7.55)	1.96 (0.51–7.53)

^a^Adjusted for Age Group, Sex, Race/Ethnicity, Comorbidities (≥ 2 vs. 0–1), Vaccination, Variant Period, days from symptom onset to hospital admission.

^b^Antiviral treatment includes remdesivir, bamlanivamab/etesevimab, or casirivimab/imdevimab.

### Anti-nucleocapsid antibody

Anti-nucleocapsid antibody results were available for a convenience sample of 187 out of 456 patients (41.0%), with specimens collected for 125 patients 0–14 days after illness onset, for 25 patients 15–28 days after onset, and for 52 patients outside these time periods. Participants with available results were similar to others included in the analysis (Supplementary Table 5). Anti-nucleocapsid antibodies varied between patients and by severity; low antibody titers were rare more than 14 days after illness onset (Table 4), and, by days 15–28, antibody levels were higher among severe patients [median (IQR) = 60.0 (39.0–83.0)] than non-severe patients [median (IQR) = 25.0 (12.0–54.0); *p* = 0.024] (Supplementary Fig. 2). Sparse data precluded inclusion of anti-nucleocapsid antibody detection in the multivariable model.

## Discussion

In this study we have characterized the demographic, clinical and laboratory aspects of COVID-19 among a population that experienced a disproportionate impact of the pandemic. Among the patients admitted with COVID-19, approximately 1 in 2 received supplemental oxygen, 1 in 3 received high-flow oxygen, and 1 in 10 died during their hospitalization. Our analysis is consistent with other reports of substantial early impact of COVID-19 in Louisiana. Together with evidence from other studies, our findings suggest that several factors may have been important in explaining the high case-severity in this cohort.

We found that patients with COVID-19 requiring high-flow oxygen were more likely to be older, which is consistent with other studies^7^. Nevertheless, approximately 66% of patients hospitalized with severe COVID-19 were younger than age 65, indicating that other factors were also important. Among all patients included in our analysis, 95% had underlying health conditions, and 80% had multiple comorbidities. Patients most frequently had cardiac disease, obesity, and diabetes mellitus, each of which are risk factors for severe outcomes from COVID-19^12^. Notably, we found that comorbidity was reflected in hospitalized cases, whether or not they received high-flow supplemental oxygen. Since we conditioned the analysis on hospital admission, comorbidity among non-severe cases might reflect an increased likelihood of admission, leading to potential collider bias^20^. A lower threshold for admission with underlying health conditions among patients without severe COVID-19 might explain why we did not find overall differences in underlying conditions by illness severity. Our finding that patients with more than two underlying conditions tended to be admitted more rapidly than other patients is suggestive of this. In view of these considerations, the lack of an overall difference in comorbidity by severity does not negate the importance of comorbidities in driving case-severity. Instead, the high prevalence of known risk factors suggests these factors were important drivers of adverse outcomes.

Among patients included in our analysis, 60.5% had Black non-Hispanic race and ethnicity. This proportion is slightly higher than that of inpatients documented to have COVID-19 in the participating hospitals (approximately 50%), and is similar to the proportion reported for New Orleans in the U.S. census (58%)^21^. Consistent with previous analyses, we did not find a difference in case-severity by race and ethnicity among hospitalized patients^5,22^. However, Black race was associated with an increased risk of hospitalization with COVID-19 in Louisiana after adjusting for comorbidity and socioeconomic status^5^, and this elevated risk might reflect an array of other factors, including those related to accessing care^6^.

Compared with non-severe hospitalized patients, we found that those requiring high-flow supplemental oxygen were more likely to be admitted greater than five days after symptom onset. This suggests that delayed access to healthcare might have contributed to adverse outcomes. In our analysis, patients received high-flow oxygen a median of 8 days after symptom onset, and inpatient deaths occurred a median of 24 days after illness onset. Severe COVID-19 typically progresses over 1–2 weeks^23^, and patients who were admitted more than five days after illness onset were likely to have more severe illness by the time of presentation. We also found that patients with severe illness were more likely to have received treatment with remdesivir, dexamethasone, or other non-antiviral treatment (baricitinib or convalescent plasma). Since patients who met criteria for severe illness were likely to have been unwell at presentation, this is likely to reflect more treatment for patients presenting with more advanced disease, rather than any effect of treatment on severity; such an interpretation is supported by other evidence from other studies^24^.

Only 5% of hospitalized patients had completed a primary COVID-19 vaccine series during the period of analysis. This low proportion is likely to reflect both low vaccine coverage early in the pandemic, and an increased risk of COVID-19 if unvaccinated^25^. Similarly, approximately 20% of the U.S. population were estimated to have had prior infection during the period of analysis^26^. Since infection-induced immunity confers substantial protection against severe illness, patients admitted with COVID-19 would be expected to have a lower prevalence of prior infection during the period of analysis^27^. Although we did not have baseline serology results, low antibody titers during 0–7 days is consistent with a low prevalence of prior infection in the cohort.

Our finding that 43% of patients did not have a positive anti-nucleocapsid result within 14 days of illness onset is consistent with other evidence that it can take up to 14 days or longer for new antibodies to develop^28^. Since a similar proportion of patients with severe and non-severe disease had evidence of seroconversion, we did not find evidence that severe disease reflected inadequate immune responses. However, our modeled estimates were limited by sparse data.

Among patients with available SARS-CoV-2 RT-PCR results, we found that severe COVID-19 was associated with a lower cycle threshold value in saliva specimens that were collected before any antiviral treatment was started. Cycle threshold values reflect the number of RT-PCR amplification cycles needed to detect viral RNA in a specimen, and are inversely related to the level of viral RNA; lower Ct values therefore imply the presence of higher RNA levels. Higher cycle threshold values over time are likely to reflect declining viral load after initial infection^29,30^. Lower cycle threshold values among patients with severe disease is broadly consistent with evidence of higher viral load in severe illness, after adjusting for other characteristics^15,17,19^. Detection of SARS-CoV-2 in saliva may reflect involvement of the oral cavity^31^. Previous studies have found similar detection of SARS-CoV-2 RNA in nasal and saliva specimens early after symptom onset^32,33^, although with differences in cycle threshold that may reflect differences in specimen collection^33,34^. Our findings were similar when restricted to patients with paired saliva and nasal specimens on the same day. However, data were sparse for paired specimens, and reasons for an association with saliva but not nasal specimens is unknown. Our findings of an association between case-severity and lower Ct value in saliva are consistent with those of others, who have reported that abundance of SARS-CoV-2 RNA in saliva was significantly higher in patients with risk factors for severe COVID-19, correlated with more severe COVID-19, and was superior to nasopharyngeal viral load as a predictor of mortality^35^. We did not find an association between lower cycle threshold and severity after treatment that might lower the viral load^36,37^, possibly because patients with severe illness were also more likely to received such medications, thereby masking differences in viral load.

Before considering implications of our analysis, several strengths and limitations need to be considered, in addition to those listed above. First, although we provided a detailed description of more than 500 patients with severe COVID-19, for some analyses we were limited by sparse data, resulting in wide confidence intervals. Second, our capture of potential confounding factors was incomplete, which might lead to residual or unmeasured confounding in multivariable analyses. Third, although we found a relatively high mortality among hospitalized patients, we may have underestimated deaths that occurred in the community or that did not meet our definition of ‘severe illness’. For example, two patients who died without meeting this definition might have had extrapulmonary manifestations of infection^2^. Fourth, for analysis purposes we used a relatively low threshold (≥ 6 L/min) to determine severity based on oxygen level, limiting comparability with some other studies that have used 10–15 L/min as a threshold, and with guidelines that define severe illness based on oxygen saturation rather than supplemental flow^38,39^. Lastly, generalizability of our findings to other populations may be limited. Patients included in the analysis had similar overall demographic characteristics to other patients with COVID-19 in participating hospitals, but might have differed from patients admitted to other hospitals in the New Orleans area. Similarly, although overall patient characteristics were similar by availability of laboratory results, patients with laboratory data might be considered as a convenience sample within the main cohort. Overall, our scope was limited to analysis of patients who were hospitalized before widespread transmission of the Omicron SARS-CoV-2 variant and its subvariants.

Since predominance of the Omicron variant, average case-severity of SARS-CoV-2 infection has become milder^3^, both because of increased immunity from vaccination and infection^40^, and because of lower virulence compared with the Delta SARS-CoV-2 variant and ancestral variants^13^. Nevertheless, severe infections and deaths have continued to occur, both in individuals with and without clear risk factors. In our analysis, substantial comorbidity coupled with late presentation in an unvaccinated population are likely to have contributed to the high case-severity. Our study is relevant both in highlighting a patient population who experienced a disproportionate burden of COVID-19, and in describing severe COVID-19 in this group. Our findings of a correlation between severe illness and low cycle threshold in saliva may support the use of saliva PCR tests as a potential alternative to nasal PCR in the inpatient setting, though more work is needed to explore this association. To prepare for future epidemic and pandemic threats, our findings support broader efforts to address underlying inequalities and strengthen access to healthcare access and resilience of health systems^6,41^.

## Methods

### Setting and study participants

We conducted the cohort study at two healthcare systems in New Orleans, Louisiana—Tulane Medical Center and University Medical Center. Participants were eligible for inclusion if they were hospitalized at either site with a positive SARS-CoV-2 clinical nucleic acid amplification test (NAAT) result (from the participating health system or elsewhere), and if verbal informed consent was obtained. This study was reviewed and approved by the Tulane University School of Medicine Institutional Review Board and was performed in accordance with relevant guidelines (see 45 C.F.R. part 46; 21 C.F.R. part 56). Informed consent was obtained from all participants and/or their legal guardians. Among participants enrolled in the overall study, participants were included in the current analysis if they were admitted with COVID-19–associated symptoms and had a recorded date of admission and date of discharge (if survived). Participants were excluded from the analysis if they had enrolled in a clinical trial of medications used to treat COVID-19.

### Research specimen collection and testing

For a subset of participants determined by convenience, we collected additional nasal specimens, saliva specimens, or both. Nasal swabs were collected by insertion of collection swab directly into one nare followed by rotation, then repetition in other nare. Saliva was collected by asking subject to spit directly into sample collection tube containing virus transport medium (either 1xPBS with antifungal or OMNIGene oral (OraSure), depending on supply chain availability during pandemic). All samples were placed on wet ice for transport then storage at -80 °C, for later batched aliquoting, virus RNA extraction, and qRT PCR. Nasal swab fluid and saliva were processed for virus RNA using QIAamp Viral RNA Mini Kit (Cat. 52906) according to manufacturer’s instructions. Extracted RNA was tested for SARS-CoV-2 qRT-PCR following CDC protocol (N1, N2, RNase P primers), and including a standard curve (see Supplemental Methods).

For a subset of participants determined by convenience, we collected serum specimens for serologic analyses; this subset was overlapping to those with research respiratory specimens. Samples were aliquoted then stored at − 80 °C until testing for antibody. We performed testing for SARS-CoV-2 anti-nucleocapsid antibody using the reSARS™ CoV-2 (N) IgG enzyme-linked immunosorbent assay (ELISA) test from Zalgen Labs following manufacturer’s recommendations (see Supplemental Methods).

### Abstraction of medical information

Using a standardized tool, we abstracted additional information that had been collected as part of routine clinical care, including demographic information (age, sex, race, ethnicity), pre-COVID-19 medical history, pre-COVID-19 medications, SARS CoV-2 vaccination status, COVID-19-like symptoms, oxygen requirements, mortality, and medications administered during inpatient admission.

### Variable definitions

For analysis purposes, we considered a participant to have ‘severe’ COVID-19 if they received ≥ 6 L/min of supplemental oxygen, including via high-flow nasal cannula, non-invasive positive-pressure ventilation, or mechanical ventilation; this included patients who subsequently died during hospitalization. We used a cutoff of ≥ 6 L/min, as that is used in the participating hospitals as a threshold for initiation of high-flow nasal oxygen; we considered this level of support or more severe disease to approximate a score of 5–8 using the WHO scale^42^. We considered participants to have non-severe illness if they were hospitalized with COVID-19 but did not meet the definition of ‘severe’, and were discharged. We considered patients to have died from COVID-19 if death occurred during hospital admission. Patients who died without receipt of ≥ 6 L/min of oxygen were excluded from the analysis, since such patients were considered to have a potential alternative cause of death.

We defined the Delta-predominant period as July 1, 2021 through the end of the study period (September 2021), based on estimated national predominance^43^. We considered patients to be ‘vaccinated’ if they had received a 2nd mRNA COVID-19 vaccine dose or single dose of the Janssen COVID-19 vaccine at least 14 days before the date of symptom onset. We categorized treatment for COVID-19 as remdesivir, anti-spike monoclonal antibody (e.g., casirivimab/imdevimab, bamlanivimab/etesevimab), dexamethasone, anti-inflammatory monoclonal antibody (baricitinib, tocilizumab) or convalescent plasma; for analysis purposes we considered ‘antiviral medication’ to include remdesivir and anti-spike monoclonal antibodies, based on anticipated effects on viral load^36,37^. We analyzed laboratory values as continuous and, for simplicity, by whether they were greater or less than the median. Additionally, we limited analysis of cycle threshold (Ct) values to the first available nasal specimen and the first available saliva specimen collected during 0–7 days after symptom onset, since viral load typically declines after this period. We categorized SARS-CoV-2 anti-nucleocapsid antibody assay results by whether the specimen was obtained within 14 days after illness onset, since new seroconversion usually occurs within this period, or whether obtained during 15 to 28 days after onset. Underlying health conditions were categorized based on diagnoses listed in the patient’s medical record (Supplementary Table 1).

### Statistical analysis

To assess patient characteristics associated with severe disease, we compared demographic, clinical and laboratory characteristics by severity. In unadjusted analyses, we used the Student’s t-test or Wilcoxon rank sum test to compare continuous variables and the chi squared test to compare categorical variables. We used logistic regression to compare the patient characteristics by severity in a multivariable model, using broader categories to account for sparse data, and limiting treatment to receipt during 0–7 days after illness onset and before receiving ≥ 6 L/min of oxygen. Multivariable models included covariates that were hypothesized to be associated with severe illness (age group, sex, race/ethnicity, comorbidity, vaccination status, days from symptom onset to admission). In addition, we used a hierarchical approach to limit each model to covariates that were considered to be distal to each exposure of interest^44^. Multivariable assessments of the association between Ct value and severity were stratified by specimen collection before or after receipt of remdesivir or anti-spike monoclonal antibodies that might be expected to suppress viral load. All statistical analyses were performed using SAS Enterprise Guide 9.4 (SAS Institute, Inc., Cary, North Carolina).

### Data analysis

The analysis was planned by Ian D. Plumb, Claire Midgely, and Dahlene Fusco with input from other coauthors. Data preparation and analysis was conducted by Dahlene Fusco, Matthew McCullough and Jade James-Gist. Dr. Fusco had full access to all the data in the study and takes responsibility for the integrity of the data.

### Ethical approval

This study was reviewed and approved by the Tulane University School of Medicine Institutional Review Board, and was performed in accordance with relevant guidelines (see 45 C.F.R. part 46; 21 C.F.R. part 56). Informed consent was obtained from all participants and/or their legal guardians.

### Supplementary Information

Supplementary Information.

## Data Availability

Data presented in these analyses available in supplementary material or upon request to corresponding author.
