# Supplementary material for: Clinical and laboratory characteristics of patients hospitalized with severe COVID-19 in New Orleans, August 2020 to September 2021
Source: Sci Rep. 2024 Mar 19;14:6539. doi: 10.1038/s41598-024-57306-5 (PMC10951213; doi:10.1038/s41598-024-57306-5)
Supplement: Supplementary file 1 — Supplementary Information. [file 41598_2024_57306_MOESM1_ESM.docx]

**SUPPLEMENT**

**Supplementary Methods**

*SARS CoV-2 qRT PCR method.* Extracted RNA is run for SARS-CoV-2 qRT-PCR following CDC protocol (N1, N2, RNase P primers), and including standard curve. SARS CoV-2 qRT PCR was performed on nasal and saliva RNA using Taqpath. For the SARS-CoV-2 detection qRT-PCR all reagents were thawed on ice, inverted 5 times to mix and spun down before use. All work was done on ice blocks. We prepared three mastermixes (N1, N2, RP) in 1.5mL microcentrifuge tubes by adding 8.5μL RNase-free H2O, 1.5μL primer/probe set, and 5μL Taqpath per well. The three mastermixes were mixed via pipetting 15 times. Mastermixes were equally distributed to an 8-well strip, and 15μL added to their respective wells of the 384 well plate using a multichannel pipet. Next RNA was added to each well. We added 20μL RNA to each well of an 8-well strip and used a multichannel pipet to transfer 5μL to each corresponding well. N1 was measured in duplicate, N2 and RP were both measured once. 5μL of each control; PTC, nuclease-free water (NTC), or negative sample, was added to each respective well. Next the standard was prepared. The STD8 well consisted of 13μL of concentrated ssRNA, each successive well contained 18μL of diluent (tRNA from baker’s yeast in nuclease-free water). 2μL STD8 was transferred to the STD7 well and mixed thoroughly via pipetting. This procedure was repeated to the STD6 well and so on. 5μL of standard was added to each corresponding well of the 384 well plate, in duplicate. The plate was thoroughly sealed, then centrifuged for 2 minutes at 3200 rpm. PCR was performed using a QuantStudio6. PCR properties were as follows: TaqMan reagents; standard speed; passive reference: ROX. PCR Cycling conditions were as follows: stage 1 step 1: 2 min, 25°C; stage 1 step 2: 15 min, 50°C; stage 1 step 3: 2 min, 95°C; stage 2 step 1: 3 sec, 95°C; stage 2 step 2: 30 sec, 60°C. Stage 2 was run for 40 cycles.

*SARS CoV-2 Antibody testing for anti-nucleoprotein.* The reSARS™ CoV-2 (N) IgG enzyme-linked immunosorbent assay (ELISA) test from Zalgen Labs ELISA was performed on human serum or plasma following manufacturer’s recommendations [https://zalgen.com/wp-content/uploads/PI-32R-00-ReSARS CoV-2-N-IgG-ELISA-RUO.pdf]. The Zalgen assay is a semi-quantitative detection of anti-N IgG, as control plasma (“reference standard”) is provided with a titer (arbitrary OD unit/mL) specified on the label of each control tube (64 units/mL). The reference curve control is used to prepare a five-point reference curve with 4-fold serial dilutions of the control (all batches received had a value of the reference of ~64 units/mL and therefore a range of quantification of 64 to 0.25 units/mL). To calculate sample titer in OD units/mL the sample’s duplicate values’ mean OD is plotted on the Y axis against the IgG reference in unit/mL on the X axis. Positivity was then determined using the negative control as the cut off. The calculated OD values (AU/mL) were entered into REDcap (if multiple ELISAs were run for the sample, the highest OD result was used). Samples from every available clinic encounter with each study participant were analyzed and included in the data. In addition, positive and negative plasma are provided. The duplicate (per sample) OD coefficient of variation is calculated as CV(%)=100xSD/mean. Per manufacturer’s recommendations an acceptable CV(%) is ≤ 25% per duplicate for controls/samples with an OD > 0.25. The cut-off was determined by running 10 serial samples from an immunocompromised patient with PCR confirmed COVID infection who never mounted anti-SARS CoV-2 IgG (Spike or nucleocapsid) [PMID: 34201591] and calculating the mean OD + 3SD. Anti-N IgG OD data critically relies on the reproducibility of ELISA plates’ inter-run OD values. The use of a reference plasma as a calibrator reference curve in each plate provides opportunity for standardization and allows us to compare OD values over serial runs. To further control the inter-assay reproducibility, we implemented a QA/QC program. In addition to the kit positive control, 15 randomly selected samples were introduced in ~20 ELISA plates that we ran over ~4 months. We calculated the CV of OD values of each positive control, randomly selected samples, and each point of the reference curve [5 point values]. The criteria used for reproducibility is recommended by manufacturer, requiring a duplicates’ CV≤ 25% [controls and samples are run in duplicate]. The CV of average OD of inter-(x20) run 1- of positive controls were <25%, 2- of upper-range and each dilution point of reference curve had CV (%) of 0.4 to up to 9%. If samples had intra-assay duplicates > 25%, samples were run in duplicate to quadruplicate in subsequent run. For those, samples of OD≤ 64 units/mL nearly all repeat OD values had inter-run CV≤ 25%. The finding of an inter-run CV> 25% for 3 samples helped us detect transcription errors in visit / sample date. Undiluted samples with estimated OD values between 64 and 128 units/mL had inter-assay run CV≤ 25%. Samples with OD values > 128 units/mL undiluted had CV>> 25% and were resulted with a capped OD value of 128 units/mL. Negative controls had CV of 28%. Of note we tested assay cross-reactivity with anti-S IgG with a sample of the immunocompromised patient after Regeneron anti-S Ab monoclonal Ab infusion. We compared OD values of a patient with serial samples collected March, April, May of 2020, Jan Aug. and Nov of 2021 and Feb. of 2022 and plotted OD on the y axis vs time on the x axis. The curve of decline confirms the accuracy of the OD values and suggest a Cut-Off defining sero-reversion. We also controlled the rebound of anti-N after sero-reversion and re-infection as well as the absence of re-bound after spike mRNA vaccine booster.

**Supplementary Table 1: Characteristics of participants included in the study, compared with all patients documented to have COVID-19 in participating hospitals**

|  | **No. inpatients with characteristic /**  **No. eligible (%)** | **No. patients with characteristic /**  **No. in analysis (%)** |
| --- | --- | --- |
| **Age in years** |  |  |
| 18-64 | 1101 / 1651 (66.7%) | 326 / 456 (71.5%) |
| ≥65 | 419 / 1651 (25.4%) | 130 / 456 (28.5%) |
| Unknown | 124 / 1651 (7.5%) | 0 / 456 (0.0%) |
| **Gender** |  |  |
| Male | 882 / 1651 (53.4%) | 259 / 456 (56.8%) |
| Female | 691 / 1651 (41.9%) | 197 / 456 (43.2%) |
| Unknown | 78 / 1651 (4.7%) | 0 / 456 (0.0%) |
| **Race** |  |  |
| White | 537 / 1651 (32.5%) | 160 / 456 (35.1%) |
| Black | 830 / 1651 (50.3%) | 276 / 456 (60.5%) |
| Asian | 10 / 1651 (0.6%) | 5 / 456 (1.1%) |
| Other | 85 / 1651 (5.1%) | 12 / 456 (2.6%) |
| Unknown | 189 / 1651 (11.4%) | 3 / 456 (0.7%) |
| **Ethnicity** |  |  |
| Hispanic | 149 / 1651 (9%) | 14 / 456 (3.1%) |
| Non-Hispanic | 1343 / 1651 (81.3%) | 441 / 456 (96.7%) |
| Unknown | 1101 / 1651 (66.7%) | 1 / 456 (0.2%) |

**Supplementary Table 2: Characteristics of participants included in the *analysis*, compared with other patients enrolled**

|  | **Included in analysis** | **Not included in analysis^a^** | **P-value** |
| --- | --- | --- | --- |
| **Age group, years** |  |  | 0.408 |
| 18–44 | 112 / 456 (24.6%) | 22 / 71 (31.0%) |  |
| 45–64 | 214 / 456 (46.9%) | 33 / 71 (46.5%) |  |
| ≥65 | 130 / 456 (28.5%) | 16 / 71 (22.5%) |  |
| **Sex** |  |  | 0.135 |
| Female | 197 / 456 (43.2%) | 24 / 71 (33.8%) |  |
| Male | 259 / 456 (56.8%) | 47 / 71 (66.2%) |  |
| **Race and ethnicity** |  |  | 0.489 |
| White, non-Hispanic | 151 / 456 (33.1%) | 28 / 71 (39.4%) |  |
| Black, non-Hispanic | 275 / 456 (60.3%) | 37 / 71 (52.1%) |  |
| Hispanic | 14 / 456 (3.1%) | 2 / 71 (2.8%) |  |
| Other, non-Hispanic | 16 / 456 (3.5%) | 4 / 71 (5.6%) |  |
| **Any underlying health conditions** |  |  | 0.130 |
| 0 | 22 / 456 (4.8%) | 5 / 71 (7.0%) |  |
| 1 | 64 / 456 (14.0%) | 14 / 71 (19.7%) |  |
| 2 | 75 / 456 (16.4%) | 16 / 71 (22.5%) |  |
| >2 | 322 / 458 (62.8%) | 36 / 71 (50.7%) |  |
| **Vaccination status at symptom onset** |  |  | >0.999 |
| Did not complete primary series | 431 / 456 (94.5%) | 67 / 71 (94.4%) |  |
| Completed primary series^b^ | 25 / 456 (5.5%) | 4 / 71 (5.6%) |  |
| **Variant predominance during illness onset^c^** |  |  | 0.281 |
| Pre-Delta | 258 / 456 (56.6%) | 45 / 71 (63.4%) |  |
| Delta | 198 / 456 (43.4%) | 26 / 71 (36.6%) |  |
| **Severity^d^** |  |  |  |
| No oxygen | 48 / 456 (10.5%) | 14 / 71 (19.7%) | 0.025 |
| Oxygen, <6L/min | 234 / 456 (51.3%) | 30 / 71 (42.3%) | 0.155 |
| Oxygen, ≥6L/min | 163 / 456 (35.7%) | 26 / 71 (36.6%) | 0.886 |
| Intubated | 69 / 456 (15.1%) | 9 / 71 (12.7%) | 0.588 |
| Died during hospitalization | 60 / 456 (13.2%) | 11 / 71 (15.5%) | 0.592 |

Exclusion criteria: Enrolled in clinical trial for COVID-19 treatment, did not test positive for COVID-19, or did not receive high-flow oxygen or intubation before death.

b Completed primary series if 2+ mRNA or 1+ J&J ≥14 days before symptom onset; Did not complete primary series if <2 mRNA doses <14 days before symptom onset

c Delta variant predominance defined as July 1, 2021 through end of study period (September 2021).

^d^ Ever recorded severity level on medical record;Groups not mutually exclusive.

**Supplemental Table 3: Characteristics of participants by availability of cycle threshold results**

|  | **No result**  n/N (col %) | **Nasal or Saliva Ct result** | **P-value** |
| --- | --- | --- | --- |
| **Age group, years** |  |  | 0.411 |
| 18–44 | 14 / 65 (21.5%) | 98 / 391 (25.0%) |  |
| 45–64 | 28 / 65 (43.1%) | 186 / 391 (47.6%) |  |
| ≥65 | 23 / 65 (35.4%) | 107 / 391 (27.4%) |  |
| **Sex** |  |  | 0.032 |
| Female | 36 / 65 (55.4%) | 161 / 391 (41.2%) |  |
| Male | 29 / 65 (44.6%) | 230 / 391 (58.8%) |  |
| **Race and ethnicity** |  |  | 0.392 |
| Black, non-Hispanic | 42 / 65 (64.6%) | 233 / 391 (59.6%) |  |
| White, non-Hispanic | 18 / 65 (27.7%) | 133 / 391 (34.0%) |  |
| Hispanic | 1 / 65 (1.5%) | 13 / 391 (3.3%) |  |
| Other, non-Hispanic | 4 / 65 (6.2%) | 12 / 391 (3.1%) |  |
| **Any underlying health conditions^a^** |  |  | 0.624 |
| 0 | 2 / 65 (3.1%) | 20 / 391 (5.1%) |  |
| 1 | 11 / 65 (16.9%) | 53 / 391 (13.6%) |  |
| ≥2 | 52 / 65 (80.0%) | 318 / 391 (81.3%) |  |
| **Vaccination status at illness onset** |  |  | 0.358 |
| Did not complete primary series | 63 / 65 (96.9%) | 368 / 391 (94.1%) |  |
| Completed primary series^b^ | 2 / 65 (3.1%) | 23 / 391 (5.9%) |  |
| **Variant period of illness onset** |  |  | 0.011 |
| Pre-Delta | 47 / 65 (72.3%) | 217 / 391 (55.5%) |  |
| Delta | 18 / 65 (27.7%) | 174 / 391 (44.5%) |  |
| **Severity^c^** |  |  | <0.001 |
| No oxygen | 15 / 65 (23.1%) | 119 / 391 (30.4%) |  |
| Oxygen, <6L/min | 12 / 65 (18.5%) | 123 / 391 (31.5%) |  |
| Oxygen, ≥6L/min (not intubated) | 14 / 65 (21.5%) | 93 / 391 (23.8%) |  |
| Intubated | 5 / 65 (7.7%) | 15 / 391 (3.8%) |  |
| Died during hospitalization | 19 / 65 (29.2%) | 41 / 391 (10.5%) |  |

aAny underlying health conditions [0-14]: Cardiac disease, Pulmonary disease, Neurologic disease, Renal disease, Liver disease, Immunocompromised, Diabetes, Hematological disease, Autoimmune disease, Smoking history, Substance abuse history, Obesity, Endocrine disease, and/or Gastrointestinal disease.

b Completed primary series if 2+ mRNA or 1+ J&J ≥14 days before symptom onset; Did not complete primary series if <2 mRNA doses <14 days before symptom onset

c If ever recorded on medical record; groups not mutually exclusive.

**Supplementary Figure 1: Cycle threshold values for detection of SARS-CoV-2 using PCR, by days since onset, and by severity**


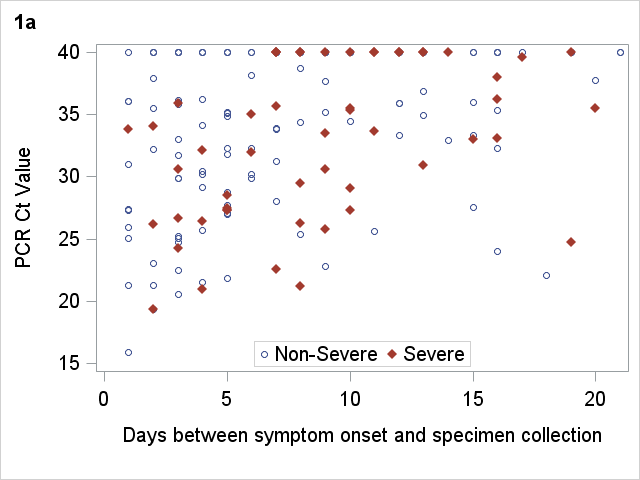

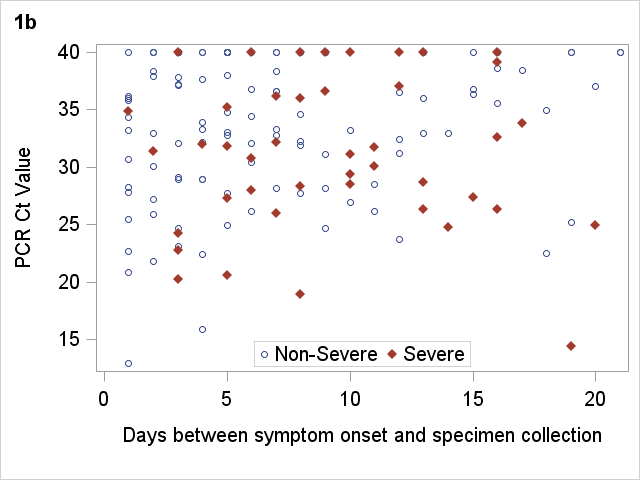


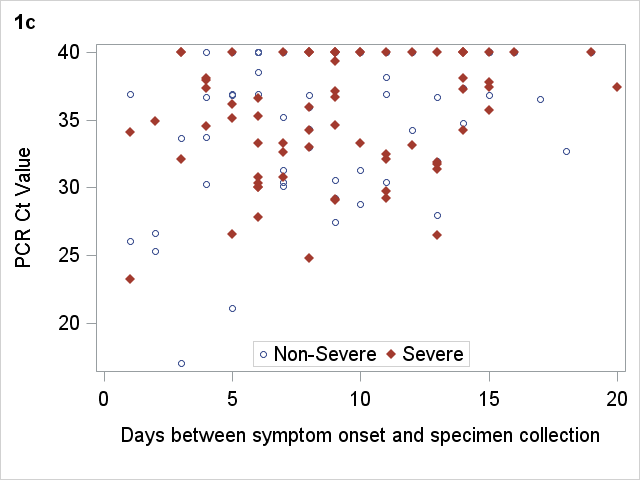

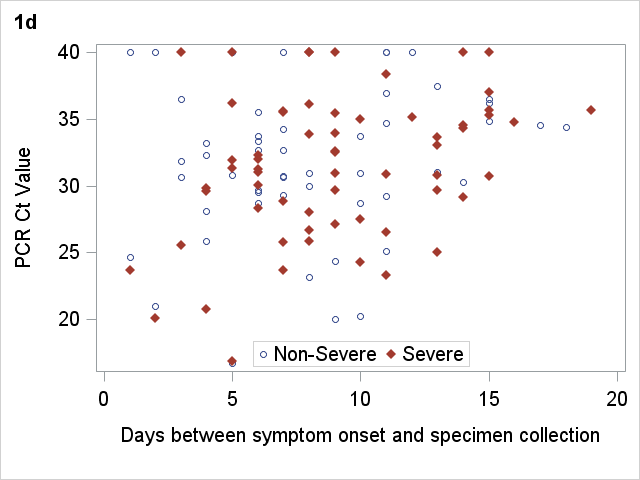


Supplemental Figure 1.

Distribution of PCR N1 cycle threshold (Ct) values by time from symptom onset to PCR specimen collection. Panels 1a and 1b represent Ct values of nasal and saliva (respective) specimens collected before or without receipt of antiviral treatment (remdesivir, casirivimab/imdevimab, bamlanivimab/etesevimab). Panels 1c and 1d represent Ct values of nasal and saliva (respective) specimens collected after receipt of antiviral treatment includes receipt of remdesivir, casirivimab/imdevimab, bamlanivimab/etesevimab).

**Supplementary Table 4: Sensitivity analyses: Models of laboratory characteristics associated with severity among patients admitted with COVID-19, August 2020 – September 2021**

|  | **No. severe / Total in category (% severe)** | **Unadjusted OR (95% CI)** | **Adjusted^a^ OR (95% CI)** |
| --- | --- | --- | --- |
| ***Model A: Restricted to paired nasal and saliva specimens collected on the same day*** | | | |
| **Nasal specimen before or without antiviral medication (0–7 days after onset)^b^** |  |  |  |
| Ct ≥32 | 6 / 44 (13.6%) | Ref | Ref |
| Ct <32 | 9 / 43 (20.9%) | 1.50 (0.58-3.87) | 1.29 (0.44-3.76) |
| **Nasal specimen after antiviral medication**  **(0–7 days after onset)** |  |  |  |
| Ct ≥32 | 14 / 30 (46.7%) | Ref | Ref |
| Ct <32 | 8 / 17 (47.1%) | 1.26 (0.40-3.91) | 1.17 (0.28-4.92) |
| **Saliva specimen before or without antiviral medication (0–7 days after onset)** |  |  |  |
| Ct ≥32 | 5 / 55 (9.1%) | Ref | Ref |
| Ct <32 | 10 / 32 (31.3%) | 4.13 (1.50-11.37) | 5.98 (1.70-21.05) |
| **Saliva specimen after antiviral medication**  **(0–7 days after onset)** |  |  |  |
| Ct ≥32 | 8 / 20 (40.0%) | Ref | Ref |
| Ct <32 | 14 / 24 (58.3%) | 1.24 (0.43-3.59) | 0.93 (0.28-3.17) |
|  | | | |
| ***Model B: Restricted to paired nasal and saliva specimens collected on the same day, before receipt of oxygen*** | | | |
| **Nasal specimen before or without antiviral medication (0–7 days after onset) ^b^** |  |  |  |
| Ct ≥32 | 4 / 39 (10.3%) | Ref | Ref |
| Ct <32 | 6 / 29 (20.7%) | 2.28 (0.58-8.98) | 1.98 (0.345-11.53) |
| **Nasal specimen after antiviral medication**  **(0–7 days after onset)** |  |  |  |
| Ct ≥32 | 6 / 8 (75.0%) | Ref | Ref |
| Ct <32 | 7 / 10 (70.0%) | 0.78 (0.10-6.32) | 0.85 (0.08-8.83) |
| **Saliva specimen before or without antiviral medication (0–7 days after onset)** |  |  |  |
| Ct ≥32 | 3 / 41 (7.3%) | Ref | Ref |
| Ct <32 | 7 / 27 (25.9%) | 4.43 (1.03-19.03) | 7.51 (1.09-51.57) |
| **Saliva specimen after antiviral medication**  **(0–7 days after onset)** |  |  |  |
| Ct ≥32 | 5 / 7 (71.4%) | Ref | Ref |
| Ct <32 | 8 / 11 72.3%) | 1.07 (0.13-8.79) | 0.91 (0.04-23.31) |
| ***Model C: Specimens collected 0–14 days after onset*** | | | |
| **Nasal specimen before or without antiviral medication (0–14 days after onset) ^c^** |  |  |  |
| Ct ≥32 | 23 / 94 (24.5%) | Ref | Ref |
| Ct <32 | 23 / 63 (36.5%) | 1.37 (0.75-2.50) | 1.42 (0.71-2.81) |
| **Nasal specimen after antiviral medication**  **(0–14 days after onset)** |  |  |  |
| Ct ≥32 | 46 / 82 (56.1%) | Ref | Ref |
| Ct <32 | 17 / 35 (48.6%) | 0.85 (0.41-1.73) | 0.81 (0.36-1.82) |
| **Saliva specimen before or without antiviral medication (0–14 days after onset)** |  |  |  |
| Ct ≥32 | 15 / 84 (17.9%) | Ref | Ref |
| Ct <32 | 20 / 55 (36.4%) | 2.28 (0.19-4.37) | 3.05 (1.46-6.39) |
| **Saliva specimen after antiviral medication**  **(0–14 days after onset)** |  |  |  |
| Ct ≥32 | 25 / 45 (55.6%) | Ref | Ref |
| Ct <32 | 30 / 54 (55.6%) | 0.87 (0.43-1.77) | 0.87 (0.40-1.92) |

a Adjusted for Age Group, Sex, Race/Ethnicity, Comorbidities (≥2 vs 0-1), Vaccination, Variant Period, and if the specimen was collected before or after the receipt of antiviral medication (remdesivir, bamlanivamab/etesevimab, or casirivimab/imdevimab).

b Specimen collected within 7 days of symptom onset, during hospitalization.

c Specimen collected within 14 days of symptom onset, during hospitalization.

**Supplementary Table 5: Characteristics of participants included in the analysis, by available anti-nucleocapsid antibody results**

|  | Patients with no anti-nucleocapsid antibody result | Patients with an anti-nucleocapsid antibody result | P-value |
| --- | --- | --- | --- |
| **Age group, years** |  |  | 0.928 |
| 18–44 | 66 / 269 (24.5%) | 46 / 187 (24.6%) |  |
| 45–64 | 128 / 269 (47.6%) | 86 / 187 (46.0%) |  |
| ≥65 | 75 / 269 (27.9%) | 55 / 187 (29.4%) |  |
| **Sex** |  |  | 0.88 |
| Female | 117 / 269 (43.5%) | 80 / 187 (42.8%) |  |
| Male | 152 / 269 (56.5%) | 107 / 187 (57.2%) |  |
| **Race and ethnicity** |  |  | 0.044 |
| White, non-Hispanic | 166 / 269 (61.7%) | 109 / 187 (58.3%) |  |
| Black, non-Hispanic | 83 / 269 (30.9%) | 68 / 187 (36.3%) |  |
| Hispanic | 6 / 269 (2.2%) | 8 / 187 (4.3%) |  |
| Other, non-Hispanic | 14 / 269 (5.2%) | 2 / 187 (1.1%) |  |
| **Any underlying health conditions^a^** | |  | 0.488 |
| 0 | 11 / 269 (4.1%) | 11 / 187 (5.9%) |  |
| 1 | 41 / 269 (15.2%) | 23 / 187 (12.3%) |  |
| ≥2 | 217 / 269 (80.7%) | 153 / 187 (81.8%) |  |
| **Vaccination status at illness onset** | |  | 0.916 |
| Did not complete primary series | 254 / 269 (94.4%) | 177 / 187 (94.7%) |  |
| Completed primary seriesb | 15 / 269 (5.6%) | 10 / 187 (5.3%) |  |
| **Variant period of illness onset^c^** | |  | 0.092 |
| Pre-Delta | 147 / 269 (54.7%) | 117 / 187 (62.6%) |  |
| Delta | 122 / 269 (45.3%) | 70 / 187 (37.4%) |  |
| **Severityd** |  |  | 0.807 |
| No oxygen | 78 / 269 (29.0%) | 56 / 187 (30.0%) |  |
| Oxygen, <6L/min | 79 / 269 (29.4%) | 56 / 187 (30.0%) |  |
| Oxygen, ≥6L/min | 63 / 269 (23.4%) | 44 / 187 (23.5%) |  |
| Intubated | 10 / 269 (3.7%) | 10 / 187 (5.3%) |  |
| Died during hospitalization | 39 / 269 (14.5%) | 21 / 187 (11.2%) |  |

a Any underlying health conditions: Cardiac disease, Pulmonary disease, Neurologic disease, Renal disease, Liver disease, Immunocompromised, Diabetes, Hematological disease, Autoimmune disease, Smoking history, Substance abuse history, Obesity, Endocrine disease, and/or Gastrointestinal disease.

b Completed primary series if 2+ mRNA or 1+ J&J ≥14 days before symptom onset; Did not complete primary series if <2 mRNA doses <14 days before symptom onset

c Delta variant predominance defined as July 1, 2021 through end of study period (September 2021).

d If ever recorded severity level on medical record; groups not mutually exclusive.

**Supplementary Figure 2: Distribution of anti-Nucleocapsid Antibody levels over time, from symptom onset to date of specimen collection, by severity**


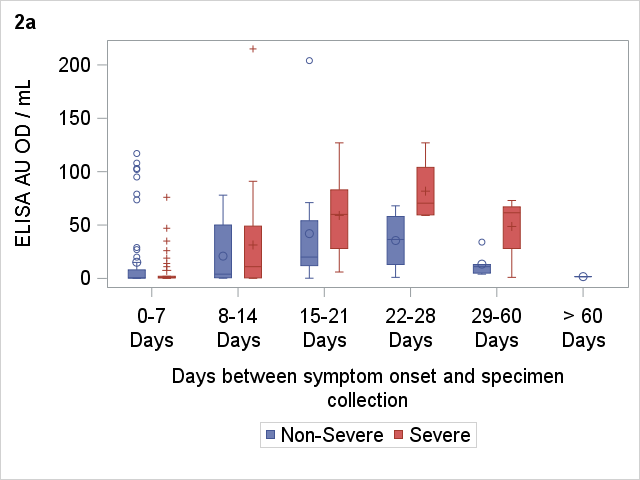

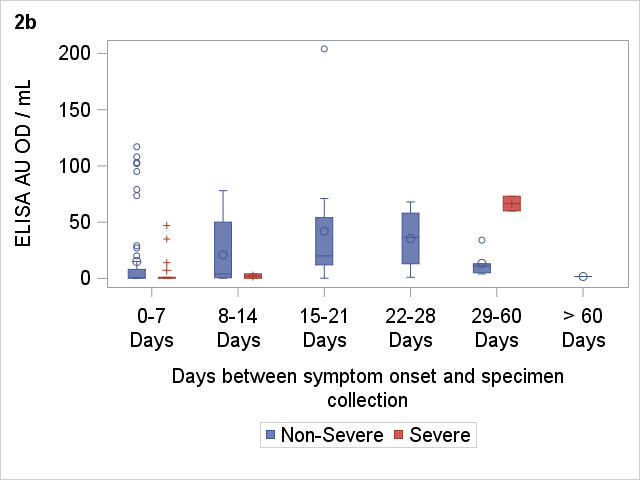


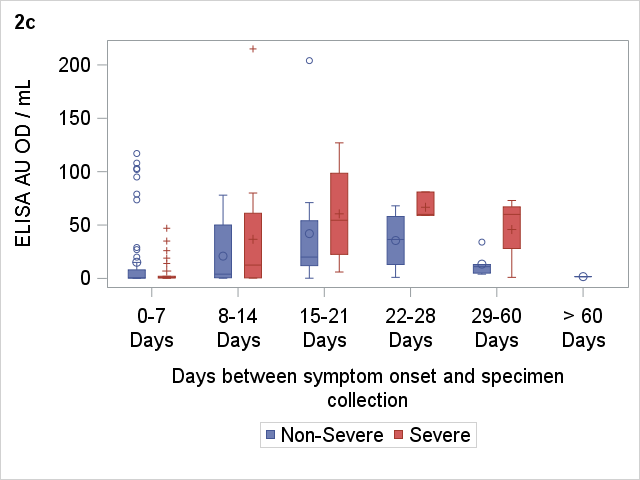

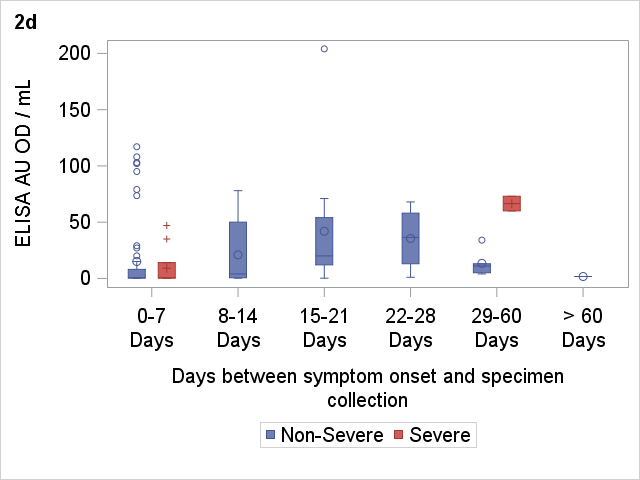


Supplemental Figure 2. Distribution of anti-nucleocapsid antibody levels (measured via enzyme-linked immunosorbent assay [ELISA] arbitrary unit optical density per milliliter [AU OD/mL]) by time from symptom onset to specimen collection during hospitalization. Panel 2a represents the distribution of antibody values collected during hospitalization, and panel 2b is restricted to specimens collected before receipt of high-flow supplemental oxygen (the analytic outcome). Panels 2c and 2d exclude patients who have died, and represent distributions of antibody values during hospitalization (2c), or during hospitalization before receipt of high-flow oxygen (2d).
